# Misalignment Decoupling and Tilt-to-Length Suppression in a Micro-Actuated Beam Steering Mechanism via Nonlinear Cyclic Modulation

**DOI:** 10.3390/mi17050587

**Published:** 2026-05-10

**Authors:** Yang Li, Changkang Fu, Hongming Zhang, Hongyang Guo, Zhiqiang Zhao, Mengyang Zhao, Ruihong Gao, Qiang Wang, Chen Wang, Caiwen Ma, Dong He, Yongmei Huang

**Affiliations:** 1 State Key Laboratory of Optical Field Manipulation Science and Technology, Institute of Optics and Electronics, Chinese Academy of Sciences, Chengdu 610209, China; liyang21d@mails.ucas.ac.cn (Y.L.); fuchangkang22@mails.ucas.ac.cn (C.F.); zhanghongming22@mails.ucas.ac.cn (H.Z.); guohongy93@163.com (H.G.); zzqiang01@163.com (Z.Z.); qiangwang@ioe.ac.cn (Q.W.); 2 University of Chinese Academy of Sciences, Beijing 100049, China; zhaomengyang20@mails.ucas.ac.cn; 3 School of Fundamental Physics and Mathematical Sciences, Hangzhou Institute for Advanced Study, University of Chinese Academy of Sciences (UCAS), Hangzhou 310024, China; 4 National Microgravity Laboratory, Institute of Mechanics, Chinese Academy of Sciences, Beijing 100190, China; gaoruihong@imech.ac.cn; 5 Xi’an Institute of Optics and Precision Mechanics, Chinese Academy of Sciences, Xi’an 710119, China; wangchen@opt.ac.cn (C.W.); cwma@opt.ac.cn (C.M.); 6 Key Laboratory of Space Precision Measurement Technology, Chinese Academy of Sciences, Xi’an 710119, China

**Keywords:** tilt-to-length coupling, nonlinear cyclic modulation, lateral misalignment, the point-ahead angle mechanism, the parasitic displacement noise

## Abstract

Tilt-to-length (TTL) coupling is a critical noise source in high-precision interferometric measurements, particularly in systems involving angular actuation and beam steering. This paper proposes a nonlinear cyclic modulation method to identify lateral misalignment and suppress the associated TTL coupling. By applying controlled sinusoidal angular excitation and evaluating the complex modulus ratio between the optical path difference (OPD) and the beam angle at the modulation frequency, the TTL noise induced by the point-ahead angle mechanism (PAAM) is separated and quantified in the frequency domain. Experimental results demonstrate that lateral offset correction reduces TTL noise by 94%, corresponding to a suppression factor of 15.5 and enabling pointing control better than 21 µm/rad. Meanwhile, the parasitic displacement noise of the PAAM is reduced from 10 pm/Hz^1/2^ to below 4 pm/Hz^1/2^. These results validate the effectiveness of the proposed modulation-based identification framework and demonstrate its applicability to precision interferometric systems.

## 1. Introduction

Micro-actuated beam steering mechanisms are widely employed in precision optical systems, including laser communication terminals, interferometric metrology platforms, and scanning-based measurement instruments. A representative example is the point-ahead angle mechanism (PAAM) used in space-based gravitational wave detection [1]. In such systems, errors in optical path alignment, mechanical stresses, beam pointing deviations, structural jitter, and wavefront aberration all couple together, resulting in changes in the longitudinal path length sensing (LPS) signal at the interferometer output [2]. This cross-coupling interference between angular and LPS is referred to as geometric and non-geometric tilt-to-length (TTL) coupling noise [3,4], which is the second largest noise source in space-based gravitational wave detectors after shot noise, fundamentally limiting the sensitivity of pm-level displacement measurements [5,6,7].

In recent years, researchers have not only analyzed the noise mechanisms and mathematical models of TTL but also proposed various methods to suppress it. In 2017, M.D. Lieser [8] analyzed the TTL coupling noise introduced by static beam angular deviations and proposed suppression methods using dual-lens and quadruple-lens imaging systems. When the quadrant photodiode (QPD) is located at the pupil plane of the imaging system, the two imaging systems successfully suppress the TTL coupling noise to a pointing control level below ±25 μm/rad. However, the dual-lens imaging system is sensitive to beam parameters, while the quadruple-lens imaging system has a more complex design. In 2022, S. Paczkowski used differential wavefront sensing (DWS) to estimate the TTL contribution and subsequently subtract TTL noise from the time-delay interferometry (TDI) data stream [9]. The proposed noise minimization method was validated using the laser interferometer space antenna (LISA) simulator on 100 data sets, achieving TTL noise estimation with an accuracy superior to ±0.1 mm/rad, resulting in residual TTL noise lower than the current estimates of other noise sources. However, this method requires the estimation of 24 TTL noise coefficients, which leads to a high computational complexity. In 2023, M.-S. Hartig derived the total TTL theoretically and conducted numerical simulations, providing a detailed analysis of the noise mechanisms and analytical models of TTL [3,4]. By rotating the interference beam around the longitudinal displacement pivot, it was found that geometric TTL and non-geometric TTL can cancel each other out. However, when the beam waist radii of the two beams differ, the total TTL does not become zero. And a special solution was proposed to shift the beam’s rotation center from the detector surface longitudinally to the beam waist position using the imaging system, which allows for cancellation once again. In 2024, X. Wang proposed a TTL post-processing method based on a second-order estimation model, achieving a reduction of the average residual TTL noise to below 8.2%, with higher precision compared to the first-order model [10]. However, under the assumption of considering only the transmitter jitter, the first-order model has 12 parameters, while the second-order model has 30 parameters. Meanwhile, G. Wanner used all three Michelson observables to fit the TTL coefficients, addressing the correlation between the coupling coefficients of the transmitter and receiver jitter in each TDI Michelson observable, and effectively reduced the total TTL noise in the LISA mission [11]. In 2025, L.-J. Zhao employed a Monte Carlo method for wavefront generation and tolerance analysis [5]. The study analyzed the impact of various aberrations in the far-field wavefront of the telescope on non-geometric TTL. It was demonstrated that correcting for coma during the telescope design phase can effectively suppress TTL noise, particularly the correction of primary coma and field-cubic coma, which enables TTL noise to converge rapidly.

Overall, geometric TTL noise suppression methods primarily include lens imaging systems based on Fermat’s principle [6,12] and TDI-based post-processing subtraction techniques [13,14,15,16], while non-geometric TTL noise suppression approaches mainly involve aberration parameter optimization and total TTL balancing strategies. This paper focuses on suppressing geometric TTL noise in PAAM systems, which is described as the path length variation perpendicular to the mirror surface caused by the coupling of lateral shift and mechanism deflection angle when the center of the incident spot deviates from the mirror center. Therefore, the above imaging systems can be used to image the incident point of the beam to the mirror center of PAAM, thereby achieving suppression of TTL noise. However, methods based on TTL coupling coefficient estimation and TDI post-processing cannot be used to reduce the TTL noise associated with PAAM, because the interferometer’s LPS signal contains not only TTL noise introduced by the lateral shift and angular jitter of PAAM but also includes longitudinal jitter errors of the mechanism, longitudinal jitter errors of the rotation axis, and DWS readout errors. To achieve space-based GW detection in the 0.1 mHz to 1 Hz low-frequency band, LISA allocates a pointing jitter budget for PAAM of less than 10 nrad/Hz^1/2^. The path length noise introduced by the mechanism’s tilt can be suppressed to below 1 pm when the lateral shift is controlled within 0.1 mm. But to date, there have been no detailed literature reports on high-precision calculation and quantification of lateral shifts.

Therefore, this paper proposes a TTL suppression method based on nonlinear cyclic modulation. By constructing a heterodyne interferometric optical system, the lateral offset between the incident beam spot and the PAAM mirror center is decoupled and compensated. The structure of this paper is organized as follows. Section 2 introduces the concepts of the point-ahead angle (PAA) and the point-ahead angle mechanism (PAAM), with particular emphasis on establishing a multi-parameter TTL noise model associated with the PAAM. Section 3 derives the suppression principle of PAAM-induced TTL noise. By applying cyclic nonlinear angular modulation to the micro-actuated steering mirror, the motion-induced errors of the PAAM are converted into distinct frequency components. Based on the complex modulus ratio of the modulated sinusoidal signals in the frequency domain, a lateral misalignment identification and suppression strategy is developed, which fundamentally reduces geometric TTL coupling. Section 4 presents the experimental validation of the proposed approach. The results demonstrate that the nonlinear cyclic modulation method reduces TTL noise by 94%, achieving a suppression factor of 15.5 and enabling pointing control within 21 µm/rad. Finally, Section 5 summarizes the main conclusions of this study.

## 2. Theoretical Analysis

### 2.1. The Point-Ahead Angle Mechanism (PAAM)

**Figure 1:**
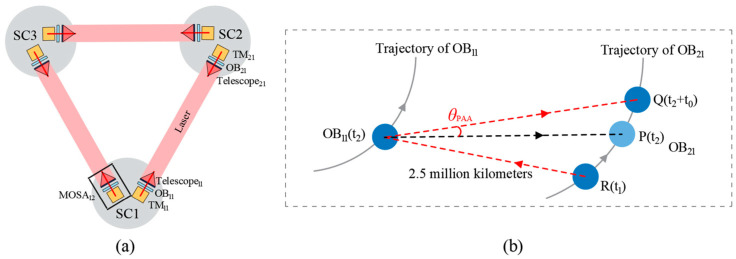
(**a**) Triangular constellation schematic of space-based gravitational wave detection; (**b**) Time-delay of beam propagation between any two spacecraft and the resulting PAA in LISA.

**Figure 2:**
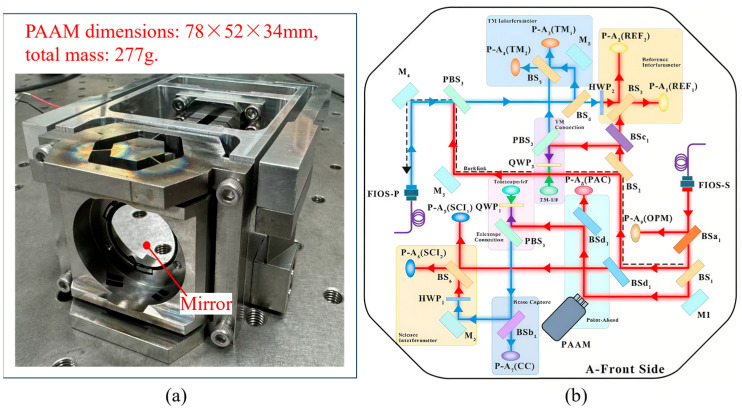
(**a**) The Point-Ahead Angle Mechanism; (**b**) The layout of the PAAM within the optical path.

A space-based gravitational wave detector consists of three spacecraft forming a giant Michelson interferometer [17,18,19], as shown in Figure 1. During long-distance laser propagation between the two spacecraft, relative along-track motion introduces a pointing offset, referred to as the point-ahead angle (PAA) [20,21]. In Figure 2, The Point-Ahead Angle Mechanism (PAAM) is designed to compensate for this offset. Essentially, the PAAM is a high-precision micro-actuated beam steering device and serves as a critical payload for enabling the laser interferometric system to reach the sensitivity required for scientific observations.

**Figure 3:**
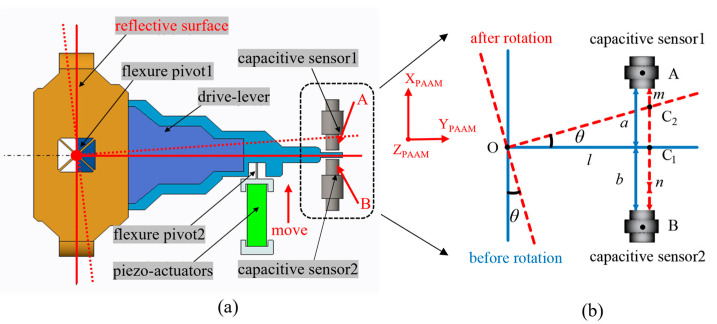
(**a**) Rotation mechanism of PAAM; (**b**) Principle of angular measurement using capacitive sensors.

The $O-X_{\mathrm{PAAM}}Y_{\mathrm{PAAM}}Z_{\mathrm{PAAM}}$ coordinate system defines the PAAM motion reference frame, in which Figure 3a shows a one-dimensional rotation along the *X*_PAAM_ direction actuated by a piezoelectric ceramic driver. The drive signal is feedback-controlled and closed-loop stabilized using angular data from a capacitive sensor [22]. Figure 3a shows the principle of angular measurement using the capacitive sensor. Points A and B denote the two probes of the capacitive sensor, with O marking the center of the PAAM mirror. When the piezoelectric actuator is unpowered, the drive rod remains horizontal, intersecting the AB line at point C_1_. When the piezoelectric actuator is energized and extends upward, the drive rod moves to point C_2_ along the AB line. The PAAM mirror is mechanically linked to the drive rod, thereby achieving precise one-dimensional tilt motion. Thus, the PAAM rotation angle can be expressed as:

(1) $$\theta =\arctan\left(\frac{\Delta h}{l}\right)=\arctan\left(\frac{n-m}{2l}+\frac{a-b}{2l}\right)$$

where Δh denotes the linear displacement of the PAAM drive rod, l represents the arm length of the PAAM, a and b denote the distances from the PAAM drive rod to capacitive sensor probes A and B, respectively, before rotation; *m* and *n* represent the corresponding distances after rotation.

### 2.2. Modeling of PAAM-Introduced TTL Coupling Effects

**Figure 4:**
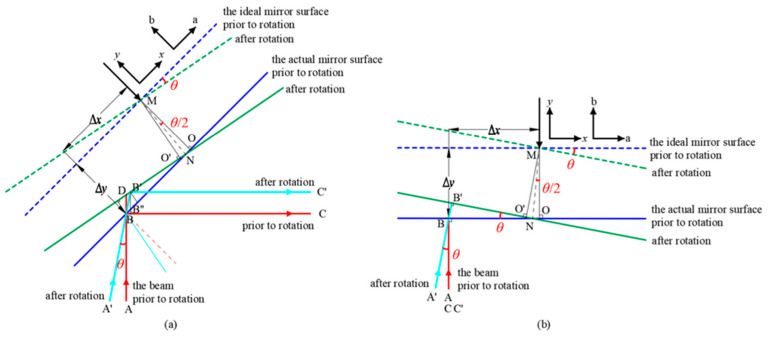
Two-dimensional schematic of the parasitic optical path difference (OPD) induced by the PAAM. (**a**) The OPD for a 45° laser beam incidence; (**b**) the OPD for a 90° laser beam incidence.

Figure 4 illustrates the parasitic optical path difference (OPD) caused by the PAAM, corresponding to PAAM-related TTL coupling [23]. Figure 4a shows the OPD for a 45° laser beam incidence, representing the actual system design for space gravitational wave detection. Figure 4b presents the OPD under 90° incidence for principle verification. In the figure, the x-axis indicates the direction along the PAAM mirror surface, while the y-axis is perpendicular to the surface. The axes a and b represent the two directions of the mirror’s rotation axis. They are separate coordinate systems. The blue and green areas represent the rotating mirror. Ray ABC and A′B′C′ correspond to laser beams from the spacecraft at different positions, exhibiting an angle *θ* caused by their movement. The incident points of both beams at the mirror are assumed to coincide at point B. The parasitic OPD is derived under the small-angle approximation as follows:

In the actual on-orbit system, the parasitic OPD induced by the PAAM is shown in Figure 4a and can be expressed as follows:

(2) $$\begin{array}{cc} {\mathrm{OPD}}_{a} & =\left(\left|{\mathrm{BB}}^{\prime }\right|+\left|B^{\prime }C^{\prime }\right|\right)-\left(\left|{\mathrm{BB}}^{\prime \prime }\right|+\left|B^{\prime \prime }C\right|\right)=\left|{\mathrm{BB}}^{\prime }\right|-\left|{\mathrm{BB}}^{\prime \prime }\right|\\ & =\frac{\left(\Delta x-\Delta y\cdot \tan\frac{\theta }{2}\right)\cdot \sin\theta }{\cos\left(\frac{\pi }{4}+\theta \right)}\cdot \left(1-\sin\theta \right)\\ & \approx \sqrt{2}\left(\Delta x-\Delta y\cdot \tan\frac{\theta }{2}\right)\cdot \sin\theta \\ & \approx \left|\mathrm{BD}\right| \end{array}$$

In the ground-based experimental system, the parasitic OPD induced by the PAAM is shown in Figure 4b and can be expressed as follows:

(3) $${\mathrm{OPD}}_{b}=2\cdot \left|{\mathrm{BB}}^{\prime }\right|=2\left(\Delta x-\Delta y\cdot \tan\frac{\theta }{2}\right)\cdot \sin\theta$$

By performing a Taylor expansion with respect to the small angle *θ* and retaining terms up to the third order, the optical path difference can be expressed as follows:

(4) $${\mathrm{OPD}}_{b}=2\Delta x\theta -\Delta y\theta^{2}-\frac{\Delta x}{3}\theta^{3}+O\left(\theta^{4}\right)$$

Thus,

(5) $${\mathrm{OPD}}_{b}\approx 2\left(\Delta x-\Delta y\cdot \frac{\theta }{2}\right)\cdot \theta$$

The multi-order response of the optical path difference arises from the coupling between angular motion and beam spot misalignment. In addition, angular jitter, PAAM translational jitter, and rotation axis jitter also contribute to parasitic optical path differences. From the above expression, the parasitic OPD is determined by eight independent variables, as listed and defined in Table 1.

**Table 1:** The descriptions and definitions of physical parameters.

Parameter	Description
θ	Static PAAM rotation angle.
Δx	Static lateral offset of the effective rotation center along the x-direction, arising from PAAM assembly misalignment (typically ±50 μm).
Δy	Static longitudinal offset of the effective rotation center along the y-direction due to PAAM alignment errors (typical range: ±1 mm).
δθ	Angular jitter of the PAAM.
δa	Lateral jitter of the rotation axis, equivalent to a dynamic variation of Δ*x*.
δb	Longitudinal jitter of the rotation axis, corresponding to translational motion along the y-direction.
δΔx	Lateral translational jitter of the PAAM along the x-direction.
δΔy	Longitudinal translational jitter of the PAAM along the y-direction.

Here, Δx and Δy represent the static offsets between the beam incidence point B and the rotational center of the mirror. These offsets couple with the angular displacement θ and the angular jitter error δθ, leading to variations in the longitudinal optical path length, which are referred to as PAAM-induced geometric TTL noise. When Δx=Δy=0, the beam incidence points B of rays ABC and A′B′C′ coincide exactly with the mirror’s rotation axis, resulting in no parasitic optical path difference (OPD = 0). It should be noted that, in this model, the mirror rotates about the Z-axis; therefore, mechanical tolerances along the Z-direction do not introduce additional optical path difference, and no OPD component arises along the Z-axis.

**Figure 5:**
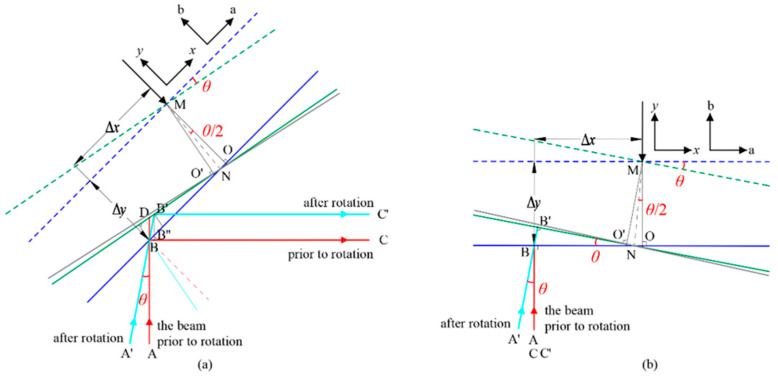
OPD induced by PAAM angular jitter δθ. (**a**) The OPD for a 45° laser beam incidence; (**b**) the OPD for a 90° laser beam incidence.

Then, the OPD induced by the PAAM rotation angle jitter (δθ) is shown in Figure 5 and described as:

(6) $$\begin{array}{cc} f_{1}^{a} & {=\mathrm{OPD}}_{a}(\theta +\delta \theta )-{\mathrm{OPD}}_{a}(\theta )=\frac{d({\mathrm{OPD}}_{a})}{d(\theta )}\\ & =\sqrt{2}\left(\Delta x-\Delta y\cdot \frac{\theta }{2}\right)\cdot \delta \theta +\Delta y\cdot \theta \cdot \delta \theta \\ & =\sqrt{2}\left(\Delta x-\Delta y\cdot \theta \right)\cdot \delta \theta \end{array}$$

(7) $$f_{1}^{b}=2\left(\Delta x-\Delta y\cdot \theta \right)\cdot \delta \theta$$

The contribution of the rotation axis lateral jitter (δa) to the OPD is given by:

(8) $$f_{2}^{a}=f_{2}^{b}=0$$

The OPD contribution associated with the PAAM translational jitter along the x-direction (δΔx) is given by:

(9) $$f_{3}^{a}=f_{2}^{a}(\delta \Delta x)+\frac{d({\mathrm{OPD}}_{a})}{d(\Delta x)}=\sqrt{2}\theta \cdot \delta \Delta x$$

(10) $$f_{3}^{b}=f_{2}^{b}(\delta \Delta x)+\frac{d({\mathrm{OPD}}_{b})}{d(\Delta x)}=2\theta \cdot \delta \Delta x$$

**Figure 6:**
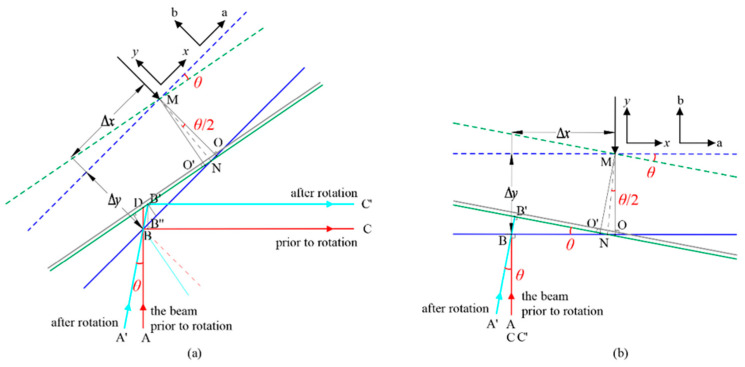
OPD induced by the longitudinal jitter of the rotation axis, δb. (**a**) The OPD for a 45° laser beam incidence; (**b**) the OPD for a 90° laser beam incidence.

The effect of the longitudinal jitter of the rotation axis ( δb ) illustrated in Figure 6. Based on the geometric relations, it can be expressed as:

(11) $$f_{4}^{a}=\sqrt{2}\delta b$$

(12) $$f_{4}^{b}=\delta b$$

The OPD resulting from the translational jitter of the entire PAAM along the y-direction, δΔy, consists of two contributions: (i) the longitudinal displacement of the mirror itself, and (ii) the corresponding longitudinal jitter of the rotation axis induced by this displacement. Substituting δa with δΔy yields:

(13) $$\begin{array}{cc} f_{5}^{a} & =f_{4}^{a}(\delta \Delta y)+\frac{d({\mathrm{OPD}}_{a})}{d(\Delta y)}\\ & =\sqrt{2}\cdot \delta \Delta y+\sqrt{2}\cdot \frac{\theta^{2}}{2}\cdot \delta \Delta y\\ & =\sqrt{2}\left(1-\frac{\theta^{2}}{2}\right)\delta \Delta y \end{array}$$

(14) $$f_{5}^{b}=f_{4}^{b}(\delta \Delta y)+\frac{d({\mathrm{OPD}}_{b})}{d(\Delta y)}=2\left(1-\frac{\theta^{2}}{2}\right)\delta \Delta y$$

As discussed above, the contribution of angular jitter errors to the parasitic OPD of the PAAM in the actual on-orbit system can be expressed as follows:

(15) $$\delta {\mathrm{OPD}}_{a}=\sum_{i=1}^{i=5}f_{i}^{a}=\sqrt{2}\left(\Delta x\delta \theta -\Delta y\theta \delta \theta +\theta \delta \Delta x+\delta b+\delta \Delta y-\frac{\theta^{2}}{2}\delta \Delta y\right)$$

By combining Equation (2), the total parasitic OPD of the PAAM in the actual on-orbit system can be expressed as follows:

(16) $${\mathrm{OPD}}_{\mathrm{tot}}^{a}={\mathrm{OPD}}_{a}+\delta {\mathrm{OPD}}_{a}$$

Similarly, in the ground-based experimental system, the contribution of angular jitter errors to the parasitic OPD of the PAAM is given by:

(17) $$\delta {\mathrm{OPD}}_{b}=\sum_{i=1}^{i=5}f_{i}^{b}=2\left(\Delta x\delta \theta -\Delta y\theta \delta \theta +\theta \delta \Delta x+\delta b+\delta \Delta y-\frac{\theta^{2}}{2}\delta \Delta y\right)$$

By combining Equation (3), the total parasitic OPD of the PAAM in the ground-based experimental system can be expressed as follows:

(18) $${\mathrm{OPD}}_{\mathrm{tot}}^{b}={\mathrm{OPD}}_{b}+\delta {\mathrm{OPD}}_{b}$$

Therefore, the relationship between the parasitic OPDs in the ground-based experimental system and the actual on-orbit system can be expressed as follows:

(19) $${\mathrm{OPD}}_{\mathrm{tot}}^{b}=\sqrt{2}\cdot {\mathrm{OPD}}_{\mathrm{tot}}^{a}$$

Here, $\delta {\mathrm{OPD}}_{a}=\sum_{i=1}^{i=5}f_{i}^{a},i=1,2,3,4,5$ and $\delta {\mathrm{OPD}}_{b}=\sum_{i=1}^{i=5}f_{i}^{b}$ represent the jitter components, while ${\mathrm{OPD}}_{a}$ and ${\mathrm{OPD}}_{b}$ refer to the static error terms. Equation (19) indicates that the parasitic optical path difference (OPD) under 90° incidence is √2 times that under 45° incidence, thereby validating the feasibility of using the ground-based experimental system to verify the performance of the actual on-orbit system.

## 3. Nonlinear Cyclic Modulation-Based TTL Suppression Method

### 3.1. Principle of the Proposed Method

According to Equation (19), the relationship between the parasitic OPD and the lateral offset can be described as follows:

(20) $$\begin{array}{cc} {\mathrm{OPD}}_{\Delta x} & ={\mathrm{OPD}}_{\mathrm{tot}}^{b}\\ & =2\left(\theta +\delta \theta \right)\Delta x+\left(\theta^{2}\delta \Delta y+\delta b-\Delta y\theta^{2}\right)+\left(2\delta \Delta y+2\theta \delta \Delta x-2\theta \Delta y\delta \theta \right) \end{array}$$

This indicates that the parasitic OPD exhibits a strictly linear dependence on the static lateral offset Δx, without higher-order coupling terms such as $\Delta x^{2}$ and $\Delta x^{3}$. Thus, this paper proposes a TTL suppression technique based on cyclic nonlinear modulation to address lateral offset errors. The principle is as follows: when the incident beam spot is offset from the PAAM mirror center by a distance Δx, a sinusoidal motion of the rotating mirror at frequency f0 induces a periodic displacement of the spot position. This results in a periodic optical path variation in the reflected beam at the same frequency, which manifests as a first-order response of the TTL signal to the PAAM rotation. By evaluating the complex modulus ratio of the optical path difference signal to the angular signal in the corresponding frequency domain, the lateral misalignment of the PAAM (Δx) can be decoupled. A precision control system is then employed to compensate for and correct the residual misalignment, thereby suppressing the TTL coupling effect.

### 3.2. Controlled Sinusoidal Excitation

The PAAM is driven into a cyclic nonlinear motion, resulting in a mechanical tilt angle that can be expressed as:

(21) $$\theta (t)=A_{0}\sin\left(\omega_{0}t\right)$$

Substituting into Equation (18), the time domain expression for the static error and jitter-induced error in the OPD signal are given by:

(22) $$\begin{array}{cc} {\mathrm{OPD}}_{b} & =-\Delta y\cdot {\left[A_{0}\sin\left(\omega_{0}t\right)\right]}^{2}+2\Delta x\cdot A_{0}\sin\left(\omega_{0}t\right)\\ & =2\Delta xA_{0}\sin\left(\omega_{0}t\right)+\frac{\Delta yA_{0}^{2}}{2}\cos\left(2\omega_{0}t\right)-\frac{\Delta yA_{0}^{2}}{2} \end{array}$$

(23) $$\begin{array}{cc} \delta {\mathrm{OPD}}_{b} & =2\Delta x\delta \theta (t)+\left(2-\frac{A_{0}^{2}}{2}\right)\delta \Delta y(t)+\delta b(t)-2\Delta yA_{0}\sin(\omega_{0}t)\delta \theta (t)\\ & +2A_{0}\sin(\omega_{0}t)\delta \Delta x(t)+\frac{A_{0}^{2}}{2}\cos(2\omega_{0}t)\delta \Delta y(t) \end{array}$$

From Equation (22), the time domain OPD contains a DC component, a fundamental frequency component, and a second-harmonic component. The fundamental frequency component, $\omega_{0}t$, is caused by the static lateral offset error, while the second-harmonic component, $2\omega_{0}t$, is influenced by the static longitudinal offset error.

It follows from Equation (23) that jitter errors convert baseband noise to frequencies near the carrier and second-harmonic components, forming sidebands that appear as noise fluctuations around these spectral components. Therefore, in the subsequent analysis, we focus on the OPD components associated with static errors.

### 3.3. Misalignment Decoupling and Tilt-to-Length Suppression

Based on the Fourier transform, the frequency spectrum of the angular signal can be obtained as follows:

(24) $${\hat{S}}_{\theta }(\omega )=i\pi A_{0}\left[\delta \left(\omega +\omega_{0}\right)-\delta \left(\omega -\omega_{0}\right)\right]$$

The frequency spectrum of the OPD signal is given by:

(25) $$\begin{array}{cc} {\hat{S}}_{L}(\omega ) & =i2\pi \Delta xA_{0}\left[\delta \left(\omega +\omega_{0}\right)-\delta \left(\omega -\omega_{0}\right)\right]\\ & \;+\frac{\pi \Delta yA_{0}^{2}}{2}\left[\delta \left(\omega +2\omega_{0}\right)+\delta \left(\omega -2\omega_{0}\right)\right]\\ & \;-\pi \Delta yA_{0}^{2}\delta \left(\omega \right) \end{array}$$

Since the signal is periodic, the single-sided power spectral densities (PSDs) of the angular signal and the OPD signal can be obtained by calculating the squared magnitude of their frequency components, respectively:

(26) $${\mathrm{PSD}}_{\theta }\left(\omega \right)=\frac{2{\left|{\hat{S}}_{\theta }(\omega )\right|}^{2}}{T}=\frac{2\pi^{2}A_{0}^{2}}{T}\delta \left(\omega -\omega_{0}\right)$$

(27) $${\mathrm{PSD}}_{L}\left(\omega \right)=\frac{2{\left|{\hat{S}}_{L}(\omega )\right|}^{2}}{T}=\frac{8\pi^{2}{\left(\Delta x\right)}^{2}A_{0}^{2}}{T}\delta \left(\omega -\omega_{0}\right)+\frac{\pi^{2}{\left(\Delta y\right)}^{2}A_{0}^{4}}{2T}\delta \left(\omega -2\omega_{0}\right)$$

Here, *T* denotes the sampling duration with a value of $T=N/F_{s}$, $F_{s}$ represents the sampling frequency, N is the data length, and $\Delta f_{s}=F_{s}/N$ denotes the frequency bin width.

The amplitude spectral density (ASD) functions are obtained as:

(28) $${\mathrm{ASD}}_{\theta }\left(\omega \right)=\sqrt{{\mathrm{PSD}}_{\theta }\left(\omega \right)}=\frac{\sqrt{2}\pi A_{0}}{\sqrt{T}}\delta \left(\omega -\omega_{0}\right)$$

(29) $${\mathrm{ASD}}_{L}\left(\omega \right)=\sqrt{{\mathrm{PSD}}_{L}\left(\omega \right)}=\frac{2\sqrt{2}\pi A_{0}\Delta x}{\sqrt{T}}\delta \left(\omega -\omega_{0}\right)+\frac{\pi A_{0}^{2}\Delta y}{\sqrt{2T}}\delta \left(\omega -2\omega_{0}\right)$$

Take the logarithm of the coordinate axis to obtain the Logarithmic Amplitude Spectral Density (LASD) functions. By extracting the fundamental-frequency component of the OPD and calculating the complex modulus ratio with respect to the steering angle at the carrier frequency, the lateral offset can be expressed as follows:

(30) $$\Delta x=\frac{{\mathrm{LASD}}_{L}\left(\omega_{0}\right)}{2{\mathrm{LASD}}_{\theta }\left(\omega_{0}\right)}=\frac{{\mathrm{ASD}}_{L}\left(\omega_{0}\right)}{2{\mathrm{ASD}}_{\theta }\left(\omega_{0}\right)}$$

Finally, a high-precision control system is employed to compensate and correct the transverse misalignment of the PAAM (Δx), thereby reducing, in principle, the physical parameters in the TTL analytical expression that are positively correlated with parasitic displacement, and suppressing TTL coupling effects. This cyclic nonlinear modulation-based TTL noise suppression method accurately captures the characteristic value of a single-frequency sinusoidal signal while eliminating disturbances from other frequency bands, offering high precision, strong robustness, and excellent anti-interference capability. Compared with TTL suppression methods based on conjugate imaging systems, the proposed method for calculating and suppressing transverse displacement does not require additional optical components and exhibits lower intrinsic noise. Furthermore, unlike the TDI post-processing subtraction strategy, this method reduces computational complexity. By directly correcting the misalignment parameters of the optical platform, it also enhances the measurement accuracy of the interferometric signal at the QPD and improves system resolution.

## 4. Experimental Validation

### 4.1. Experimental Setup

**Figure 7:**
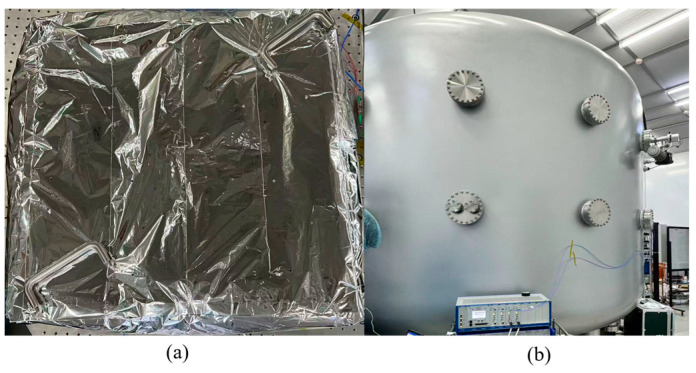
Photos of the peripheral support environment. (**a**) Optical bench with passive temperature control inside the vacuum chamber; (**b**) The vacuum chamber.

Space-based gravitational wave detection aims to observe low-frequency gravitational waves in the 0.1 mHz–1 Hz band, where temperature fluctuations represent a significant source of noise [24]. Therefore, a rigorously controlled environmental setup is required. As shown in Figure 7, the optical platform is placed inside a vacuum chamber with an independent foundation, and the atmospheric pressure is reduced to 16 Pa. Through a two-stage passive thermal control, temperature fluctuations are suppressed to below 60 mK/Hz^1/2^. The constructed ground experimental setup is used to validate the cyclic nonlinear modulation-based TTL suppression method and to measure the PAAM pointing jitter and parasitic displacement after noise suppression.

**Figure 8:**
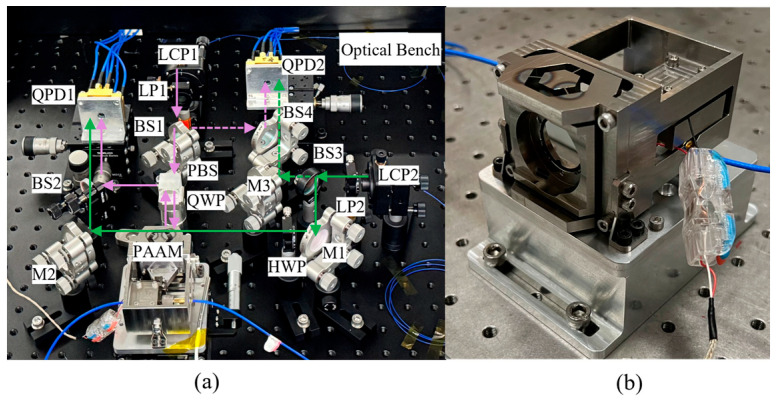
Photos of the experimental system. (**a**) The optical bench; (**b**) The PAAM mounted on a high-precision displacement stage.

Figure 8 illustrates the configuration of the experimental system, including the heterodyne interferometric optical measurement module and the PAAM closed-loop control module. The system parameters are designed as follows: the laser beam waist radius is 0.41 mm, the QPD aperture is 1.2 mm, the QPD slit width is 40 μm, the beam propagation distance in the measurement interferometer is 492 mm, that in the reference interferometer is 272 mm, and the distance between the rotational pivot and the detection surface is 122 mm for both interferometers.

As shown in Figure 8, the signal transmission procedure of the measuring interferometer is as follows: First, the measurement beam output by LCP_1_ is adjusted to P-polarized light after passing through the first Linear Polarizer (LP_1_) and transmitted to the first Beam Splitter (BS_1_) where it is split into two beams. One path is transmitted through a Polarizing Beam Splitter (PBS), then passes through a Quarter-Wave Plate (QWP), and is reflected by the PAAM. After passing through the QWP again, it is converted into S-polarization, reflected at the PBS, and redirected by BS_2_ toward the first Quadrant Photodiode (QPD_1_), forming one arm of the measurement interferometer. Meanwhile, the reference beam emitted from LCP_2_ passes through LP_2_ to become P-polarized and enters BS3, where it is also divided into two paths. The reflected beam is redirected by the first reflector mirror (M_1_), converted into S-polarization by a half-wave plate (HWP), and then reflected by the second mirror (M_2_). After transmission through BS_2_, it continues toward QPD_1_ as the second arm of the measurement interferometer. Finally, the two S-polarized beams interfere on QPD_1_, generating the measurement interference signal. Similarly, the signal transmission process of the reference interferometer can be derived.

**Figure 9:**
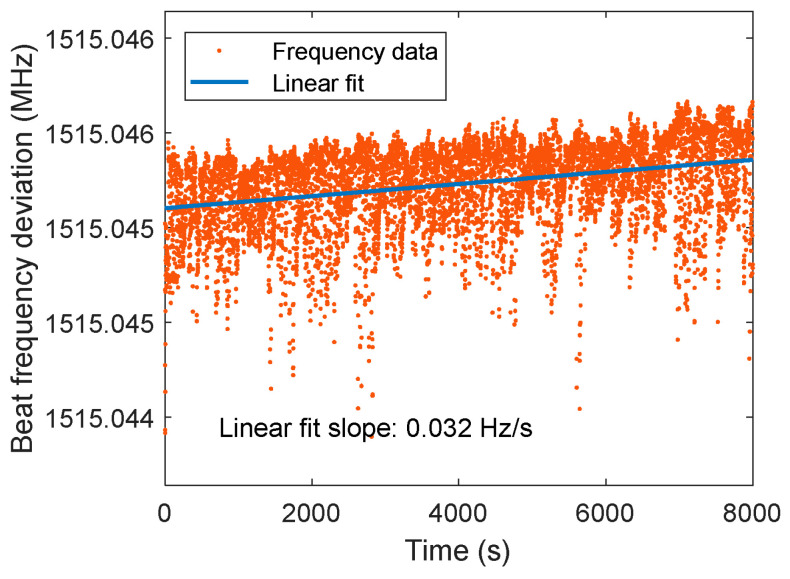
Experimental long term frequency drift data of the laser.

The basic parameters of the core equipment used in the experiment are listed in Table 2. The light source is a SLS-INT-1064-200-1 ultra-stable laser from Stable Laser Systems, USA, providing a single-frequency coherent beam with a central wavelength of 1064 nm. Figure 9 is the laser frequency sequence, in which the frequency drift is 346 Hz/3 h. A 50:50 fiber coupler splits the beam into two paths, and acousto-optic modulators (AOMs) generate two sinusoidal signals with frequencies *f*_1_ = 130 MHz and *f*_2_ = 132 MHz. The beams are transmitted to the optical platform via polarization-maintaining fibers in free-space form, forming the reference and measurement interferometer paths. The PAAM is controlled using a PI actuator from Germany. Interference signals are detected by a GD4542-20M QPD from the 44th Institute of CETC, which has a low noise-equivalent power. The QPD signals are transmitted via RF cables to a 16-channel phase meter developed in-house at the Institute of Mechanics, outputting four reference phases and four measurement phases, with a high precision of 2π μrad/Hz^1/2^ in the 0.1 mHz–1 Hz band. MATLAB R2018b is then used to calculate the PAAM angles and parasitic displacement.

### 4.2. Lateral Offset Measurement and Correction

**Figure 10:**
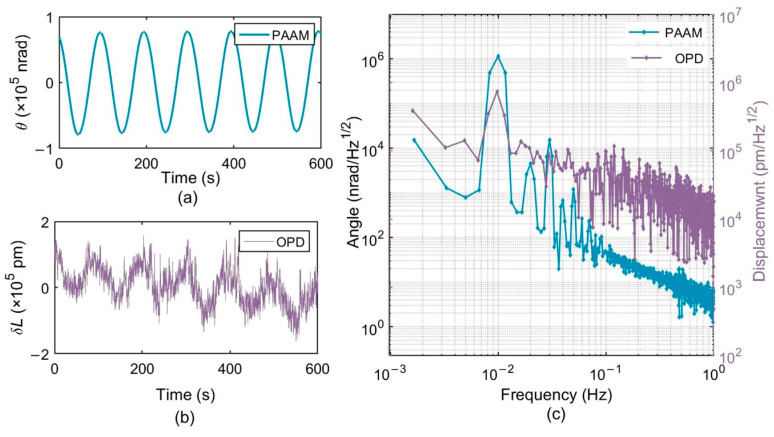
Detection signals obtained under cyclic nonlinear modulation before lateral offset suppression. (**a**) Rotation angle of PAAM measured by capacitive sensors; (**b**) Optical path difference (OPD) measured by average phase; (**c**) Frequency domain signals of PAAM angle and OPD. The abscissa represents frequency in Hz (1 mHz–1 Hz), and the ordinate represents the logarithmic amplitude spectral density (LASD) with units of nrad/√Hz for the angle and pm/√Hz for the OPD.

**Figure 11:**
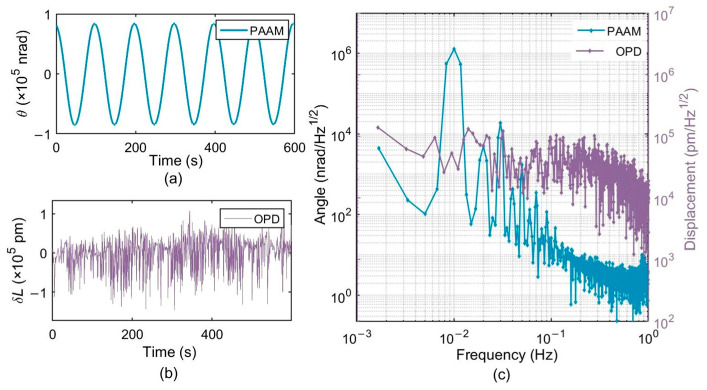
Detection signals after lateral offset quantification and suppression. (**a**) Time domain angular signal during the second PAAM drive. (**b**) Time domain OPD after lateral offset quantification and suppression, showing a clear reduction in periodic variation. (**c**) LASD frequency-domain response, including the spectra of the PAAM angle and the OPD. The abscissa represents frequency in Hz (1 mHz–1 Hz), and the ordinate represents the logarithmic amplitude spectral density (LASD) with units of nrad/√Hz for the angle and pm/√Hz for the OPD.

The PAAM was then driven to perform cyclic periodic motion in the horizontal direction at a frequency of 0.01 Hz. The phase values of the measurement QPD and the reference QPD were recorded for 10 min using a phasemeter with a sampling frequency of 20 Hz, yielding 12,000 data points. Figure 10 shows the detected signal before lateral offset suppression based on nonlinear cyclic modulation, while the corrected results after lateral offset compensation are presented in Figure 11. The carrier frequency and second-harmonic components of the OPD signals in Figure 10 and Figure 11 are induced by the lateral offset Δ*x* and the longitudinal offset Δ*y* of the PAAM, respectively. The lateral offset is decoupled using Equation (30). The sideband components are caused by jitter noise.

Table 3 summarizes the experimental results. The results show that, after correcting the lateral offset using the cyclic nonlinear modulation method, the first-order coupling between the optical path difference and the beam angle is significantly suppressed. The TTL noise is reduced by 94%, achieving pointing control with TTL coupling noise below 21 μm/rad, which is close to the theoretical value of 26 μm/rad. The proposed method provides pointing control performance comparable to that of imaging system-based techniques, while offering the advantage of requiring no additional optical components and therefore exhibiting a lower optical noise floor.

### 4.3. Residual Second-Order Response

**Figure 12:**
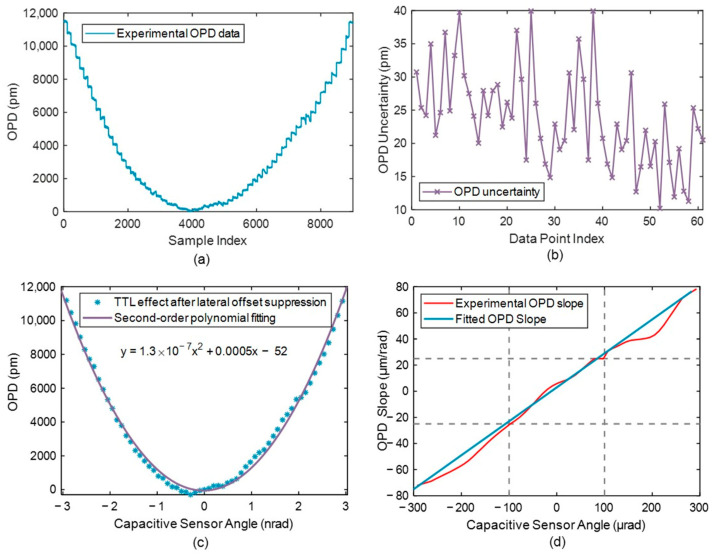
Experimental results of the PAAM-induced TTL coupling after lateral offset suppression. (**a**) Experimental OPD data. (**b**) OPD Measurement Uncertainty. (**c**) Response curves of the OPD and steering angle after lateral offset suppression. (**d**) Response curve of the OPD slope versus steering angle, illustrating the TTL coupling coefficient after lateral offset suppression.

After lateral offset suppression, the variation in OPD noise is shown in Figure 12. Figure 12a presents the experimental OPD data at different PAAM deflection angles. The PAAM was operated in closed-loop mode over an angular range from −300 to 300 μrad with a step size of 10 μrad. To mitigate environmental disturbances such as temperature drift, the duration of each step was limited to 5 s. Figure 12b shows the OPD measurement uncertainty, with an average value of 23.86 pm. Figure 12c presents the response curves of the OPD versus steering angle after lateral offset compensation, while Figure 12d shows the OPD slope at different steering angles, which is used to characterize the TTL coupling coefficient after lateral offset suppression.

The experimental results indicate that, when the lateral offset is approximately compensated, the residual deviation from the theoretical value is about 5 μm/rad. This deviation arises primarily from the following factors: (i) slight inconsistencies in the angular drive amplitude of the PAAM between different measurement runs; (ii) the suppression of most first-order TTL terms associated with the lateral offset Δ*x* based on the small-angle Taylor expansion. However, due to platform limitations, the second-order TTL coupling induced by the longitudinal offset Δ*y* has not yet been experimentally verified and will be addressed in future work, as indicated in Equation (22); (iii) system noise and thermal noise, including low-frequency temperature fluctuations, QPD clipping effects, piezoelectric actuator hysteresis, and phasemeter noise. The phasemeter used in this experiment exhibits a phase noise better than 2π μrad/√Hz (0.1 mHz–1 Hz). Using the conversion factor λ/2π, the corresponding optical path difference noise contribution from the phasemeter is estimated to be 1.06 pm/√Hz (0.1 mHz–1 Hz).

In summary, the proposed nonlinear cyclic modulation method achieves TTL suppression below ±25 μm/rad within a tilt range of ±100 μrad. It should be noted that the effective suppression range is fundamentally limited by higher-order TTL coupling terms. As the deflection angle increases, second-order and higher-order contributions become dominant, thereby constraining the suppression performance of first-order compensation methods.

Table 4 compares the proposed approach with existing TTL suppression techniques, including imaging-based methods and TDI-based strategies. The experimental results indicate that the nonlinear cyclic modulation method achieves a suppression sensitivity comparable to that of imaging system-based approaches. In contrast, the TDI-based method typically exhibits a suppression sensitivity on the order of ±0.1 mm/rad. The proposed method therefore improves the measurement precision by approximately 95%, while avoiding the need for additional optical components. Moreover, it offers advantages in terms of low intrinsic noise, high accuracy, and flexible implementation.

### 4.4. Parasitic Displacement Measurement Sensitivity

**Figure 13:**
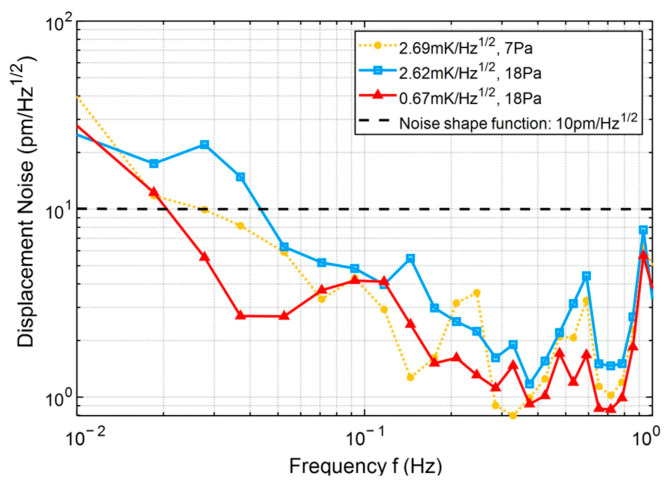
Logarithmic amplitude spectral density (LASD) of the PAAM-induced parasitic displacement before lateral offset suppression [26].

**Figure 14:**
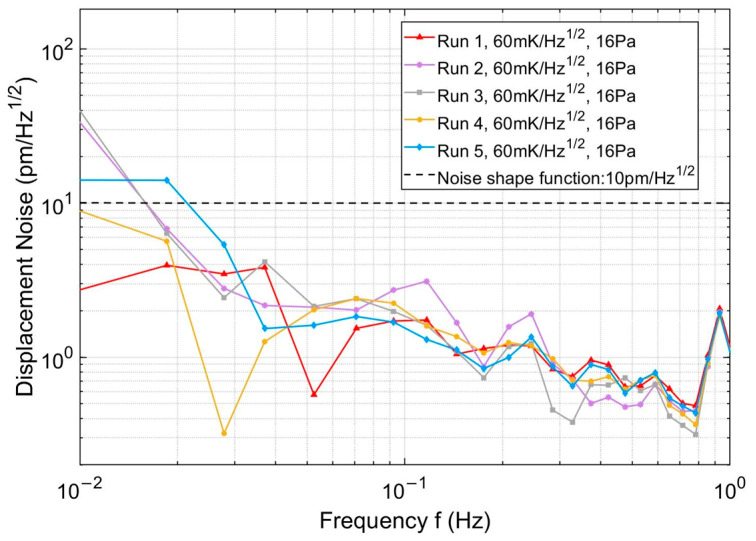
Logarithmic amplitude spectral density (LASD) of the PAAM-induced parasitic displacement after lateral offset suppression.

Figure 13 and Figure 14 show the logarithmic amplitude spectral density (LASD) of the parasitic displacement induced by the PAAM before and after lateral offset suppression, respectively. The black dashed curves in both figures represent the noise shaping function for space-based gravitational wave detection, which is described as follows [25]:

(31) $$\mathrm{NSF}\left(f\right)=\kappa_{\mathrm{NSF}}\times \sqrt{1+{\left(\frac{3\mathrm{mHz}}{f}\right)}^{4}}$$

where $\kappa_{\mathrm{NSF}}$ represents the optical measurement sensitivity.

Figure 13 presents the experimental results before lateral offset suppression. The yellow dashed curve and the blue solid curve correspond to measurements conducted under different pressure conditions. Lower atmospheric disturbance leads to reduced optical path perturbation, resulting in lower measured parasitic displacement noise. The yellow dashed curve and the red solid curve represent measurements under different temperature fluctuation conditions, where the red curve corresponds to the optimal measurement obtained after thermal equilibrium was achieved. The experimental results indicate that, within the low-frequency range of 20 mHz to 1 Hz, the parasitic displacement noise of the PAAM can reach levels below 10 pm/Hz^1/2^.

Figure 14 shows the experimental results after lateral offset suppression. Five repeated measurements were conducted under identical conditions, with a vacuum pressure of 16 Pa and a thermal stability of 60 mK/Hz^1/2^. The variation in displacement noise observed across repeated measurements is mainly attributed to the measurement uncertainty of the optical path difference (OPD), which is strongly affected by low-frequency thermal noise. Below 1 Hz, thermal noise exhibits a pronounced 1/*f* spectral behavior [27] and degrades optical measurement accuracy through two primary mechanisms [28]: (i) the refractive index of optical components is temperature-dependent; and (ii) temperature fluctuations induce thermal expansion and contraction of optical elements, leading to variations in optical path length. While the temperature-coupled noise of individual components can be characterized by their thermal expansion coefficients, temperature fluctuations in the integrated optical system are typically characterized using thermal sensors.

The red curve represents the measurement with the lowest parasitic displacement noise level among the repeated experiments in the 10 mHz–1 Hz frequency band. The results demonstrate that, within the low-frequency range of 10 mHz to 1 Hz, when the PAAM operates in closed-loop mode and is stabilized at 20 μrad, the parasitic displacement noise can be reduced to below 4 pm/Hz^1/2^.

Table 5 summarizes the parasitic displacement sensitivity and the corresponding frequency bands before and after lateral offset suppression. Even under conditions of increased thermal fluctuations and elevated atmospheric pressure, suppressing the lateral offset enhances the parasitic displacement sensitivity and extends the effective measurement bandwidth toward lower frequencies. The results indicate that the proposed nonlinear cyclic modulation method effectively compensates for and corrects lateral misalignment, thereby fundamentally reducing the coupling between lateral offset and the angular motion errors of the micro-actuated beam steering mechanism. Consequently, TTL noise is significantly suppressed, and the measurement sensitivity of picometer-level parasitic displacement in the low-frequency band is substantially improved.

## 5. Conclusions

This work systematically elucidates the geometric tilt-to-length (TTL) coupling mechanism of a micro-actuated beam steering device, namely the point-ahead angle mechanism (PAAM), by establishing a comprehensive multi-parameter mathematical model incorporating eight dominant TTL noise sources. The model clarifies the physical origins of both static misalignment and angular jitter contributions, thereby providing a rigorous foundation for identifying the primary coupling mechanisms in precision pointing systems.

Building upon this model, a nonlinear cyclic modulation-based identification and suppression strategy is proposed. Under controlled angular excitation, PAAM motion-induced errors are mapped into distinct harmonic components in the frequency domain. By evaluating the complex modulus ratio between the optical path difference (OPD) and the beam angle at the modulation frequency, lateral misalignment is decoupled and quantitatively corrected in situ. This harmonic-mapped approach eliminates the need for additional optical imaging components and avoids high-dimensional post-processing, thereby reducing optical background noise and computational complexity while enhancing measurement sensitivity.

Experimental validation in a heterodyne interferometric system demonstrates that the TTL coupling coefficient is reduced from 325 μm/rad to 21 μm/rad, corresponding to a suppression factor of 15.5. Consequently, within the 10 mHz–1 Hz frequency band, parasitic displacement noise is suppressed to below 4 pm/Hz^1/2^.

Beyond PAAM-induced coupling, the proposed nonlinear modulation framework exhibits inherent scalability and can be extended to other TTL-coupled configurations involving micro-actuated beam steering systems, including beam spot misalignment on a test mass (TM) or a tilted reflective optical element. Therefore, this study establishes a general, engineering-oriented strategy for picometer-level optical path difference measurement, providing practical guidance for precision interferometric platforms and future space-based gravitational wave detection missions.

## 6. Patents

A patent application for “A Lateral Offset Measurement System and Method Based on Sinusoidal Modulation” has been filed based on the work reported in this manuscript.

## Figures and Tables

**Table 2:** List of basic parameters for critical instruments.

Instrument Name	Instrument Model	Parameter	Value
Laser source	SLS-INT-1064-200-1	Output power	>100 mW
		Freq. stability for 1 s	$<3.5\times 10^{-15}$
		Freq. drift	<1 kHz/3 h
		Power stability	<0.1% per 3 h
QPD	GD4542-20M	Wavelength	1064 nm
		Diameter of QPD surface	1.2 mm
		Quadrant gap	≤40 μm
		3 dB bandwidth	≥20 MHz
		Noise equivalent power density @ 20 MHz	$\leq 4.5\times 10^{-12}\;W/{\mathrm{Hz}}^{1/2}$
		Output Impedance	50 Ω
		Operating voltage	±5 V
PI piezo actuator	E-619	Peak output power (<5 ms)	1200 W
		Average output current (>5 ms)	5 A
		Bandwidth	20 kHz
		Input voltage	−2 to 12 V
		Output voltage	−30 to 130 V
		Ripple (0–10 kHz)	$<2\;{\mathrm{mV}}_{\mathrm{rms}}$
		Noise (0–10 kHz)	$<20\;{\mathrm{mV}}_{\mathrm{pp}}$
Capacitive sensor	capaNCDT6500	Demodulator	DL6530
		Static resolution	$6\times 10^{-7}\;\mathrm{Hz}$
		Dynamic resolution	$4\times 10^{-4}\;\mathrm{Hz}$
		Frequency response (−3 dB)	8.5 kHz/1 kHz/20 Hz
		Measuring rate	4 × 7.8 kSa/s; 8 × 3.9 kSa/s
Phasemeter	Independently developed by the Institute of Mechanics, CAS	Frequency band	0.1 mHz–1 Hz
		phase noise level	$<2\;\pi \mu \mathrm{rad}/\sqrt{\mathrm{Hz}}$
		sampling frequency	20 MHz
		analog-to-digital converter (ADC)	AD9253

**Table 3:** Experimental TTL suppression results based on cyclic nonlinear modulation.

Suppression of the Lateral Offset	PAAM-Angle (Time Domain)	PAAM-Angle (Frequency Domain)	OPD (Frequency Domain)	TTL
Before	$7.68\times 10^{-5}\;\mathrm{rad}$	$1.14\times 10^{-3}\;\mathrm{rad}$	$7.41\times 10^{-7}\;m$	325 μm/rad
After	$8.40\times 10^{-5}\;\mathrm{rad}$	$1.26\times 10^{-3}\;\mathrm{rad}$	$5.34\times 10^{-8}\;m$	21 μm/rad

**Table 4:** Comparison of TTL Suppression Methods.

Method	Principle	Features	Suppression Results
lens imaging systems based on Fermat’s principle [8]	Using a lens assembly to image the beam spot onto the center of the QPD.	Additional optical components.	±25 μm/rad
TDI-based post-processing subtraction techniques [9]	Subtracting noise from the data stream using the estimated TTL coefficients.	Complex data processing.	±100 μm/rad
Nonlinear Cyclic Modulation (Proposed)	Misalignment Identification and Beam Spot Center Compensation.	misalignment identification; no additional optics; low intrinsic noise; high flexibility	<±25 μm/rad

**Table 5:** Comparison of parasitic displacement measurement sensitivity before and after lateral offset suppression.

Suppression of the Lateral Offset	Frequency Bands	Sensitivity
Before	20 mHz–1 Hz	10 pm/Hz^1/2^
After	10 mHz–1 Hz	4 pm/Hz^1/2^

## Data Availability

Data can be shared if relevant researchers have a reasonable need, and they can obtain the data by contacting the corresponding author.
